# Nontraumatic Hypotension and Shock in the Emergency Department and the Prehospital setting, Prevalence, Etiology, and Mortality: A Systematic Review

**DOI:** 10.1371/journal.pone.0119331

**Published:** 2015-03-19

**Authors:** Jon Gitz Holler, Camilla Nørgaard Bech, Daniel Pilsgaard Henriksen, Søren Mikkelsen, Court Pedersen, Annmarie Touborg Lassen

**Affiliations:** 1 Department of Emergency Medicine, Odense University Hospital, Odense, Denmark; 2 Department of Clinical Chemistry & Pharmacology, Odense University Hospital, Odense, Denmark; 3 Department of Anaesthesiology and Intensive Care Medicine, Odense University Hospital, Odense, Denmark; 4 Department of Infectious Diseases, Odense University Hospital, Odense, Denmark; Emory University, UNITED STATES

## Abstract

**Background:**

Acute patients presenting with hypotension in the prehospital or emergency department (ED) setting are in need of focused management and knowledge of the epidemiology characteristics might help the clinician. The aim of this review was to address prevalence, etiology and mortality of nontraumatic hypotension (SBP ≤ 90 mmHg) with or without the presence of shock in the prehospital and ED setting.

**Methods:**

We performed a systematic literature search up to August 2013, using Medline, Embase, Cinahl, Dare and The Cochrane Library. The analysis and eligibility criteria were documented according to the Preferred Reporting Items for Systematic Reviews and Meta-Analyses (PRISMA-guidelines) and The Cochrane Collaboration. No restrictions on language, publication date, or status were imposed. We used the Newcastle-Ottawa quality assessment scale (NOS-scale) and the Strengthening the Reporting of Observational studies in Epidemiology (STROBE-statement) to assess the quality.

**Results:**

Six observational studies were considered eligible for analysis based on the evaluation of 11,880 identified papers. Prehospital prevalence of hypotension was 19.5/1000 emergency medicine service (EMS) contacts, and the prevalence of hypotensive shock was 9.5-19/1000 EMS contacts with an inhospital mortality of shock between 33 to 52%. ED prevalence of hypotension was 4-13/1000 contacts with a mortality of 12%. Information on mortality, prevalence and etiology of shock in the ED was limited. A meta-analysis was not feasible due to substantial heterogeneity between studies.

**Conclusion:**

There is inadequate evidence to establish concise estimates of the characteristics of nontraumatic hypotension and shock in the ED or in the prehospital setting. The available studies suggest that 2% of EMS contacts present with nontraumatic hypotension while 1-2% present with shock. The inhospital mortality of prehospital shock is 33-52%. Prevalence of hypotension in the ED is 1% with an inhospital mortality of 12%. Prevalence, etiology and mortality of shock in the ED are not well described.

## Introduction

Focused management of acute medical patients is a cornerstone in emergency medicine. Patients in the emergency department (ED) or prehospital setting often present with heterogeneous symptoms, which challenge the initial assessment for the everyday clinician and health care worker. The initial triage of acute patients with critical illness is often supported by measurement of vital signs including systolic blood pressure (SBP) and is incorporated in many clinical guidelines as a basic part of the initial assessment of the circulation [1–3].

The presence of hypotension defined as SBP ≤ 90 mmHg is a widely accepted hallmark of possible circulatory failure and, that if persistent will lead to shock, characterised by inadequate tissue perfusion, cellular damage and metabolic changes and ultimately death unless circulation is restored [4]. Although commonly associated with it, hypotension is not synonymous to shock. Normal blood pressure can be present during shock in individuals habitually hypertensive, and normal tissue perfusion can exist among hypotensive individuals [5].

Shock is usually divided in categories according to etiology; hypovolemia-, septic-, cardiogenic- and allergic shock being the most common [4, 5]. The cause of hypotension and shock among traumatic patients is often hypovolemia due to blood loss, while the etiology among nontraumatic hypotensive patients is more disperse [4]. Correct initial assessment and resuscitation of patients with shock is crucial as prompt treatment improves the prognosis and as the optimal treatment differs depending on the cause [4]. However, the etiology of hypotension is not always clear at presentation and therefore knowledge about the clinical epidemiological characteristics might help the clinician to address this matter in the acute care setting.

We conducted a systematic review to clarify the level of evidence regarding the prevalence, etiology and mortality of unselected nontraumatic hypotensive patients with or without the presence of shock in the prehospital and Emergency Department (ED) setting.

### Objectives

The following questions were addressed:

1. What is the prevalence and etiology of unselected hypotensive patients with and without shock in the prehospital and ED setting?
2. What is the mortality of unselected hypotensive patients with and without shock in the prehospital and ED setting?

## Methods

Prior to the conduction of this systematic review a detailed protocol was developed in which the analysis and eligibility criteria were stated and documented, according to the Preferred Reporting Items for Systematic Reviews and Meta-Analyses (PRISMA) guidelines and The Cochrane Collaboration [6, 7]. See S1 Protocol and S1 Checklist in the supporting information for details.

### Eligibility criteria

We constructed a literature search involving adult hypotensive individuals (age > 15 years) with and without shock in the acute setting, by using PICOS criteria for randomized control trials (Population, Intervention, Control, Outcomes, and Study design) [6]. The acute setting was defined as an ED, or as emergency service systems (EMS, e.g. patients transported by ambulance) transporting unselected acute medical patients from a setting outside the hospital (e.g. prehospital setting) to the ED.

We defined hypotension as a SBP ≤ 90 mmHg in any unselected acute medical patient in need of medical attention. We accepted all definitions of shock as long as SBP ≤ 90 mmHg were present.

Our PICOS criteria were constructed as listed below:

Participants: Adult hypotensive (SBP ≤ 90 mmHg) patients with and without shock in the acute setting, i.e. patients assessed prehospitally or in the ED.
Intervention/exposure: All interventions regarding patients with hypotension or shock.
Comparisons: Hypotensive patients in any control group with and without shock receiving interventional treatment or standard of care treatment.
Outcome: Prevalence, Etiology and Mortality.
Study design: Randomized controlled trials (RCTs) and observational studies (cohort studies, case-control studies and cross-sectional studies).

**Fig 1:**
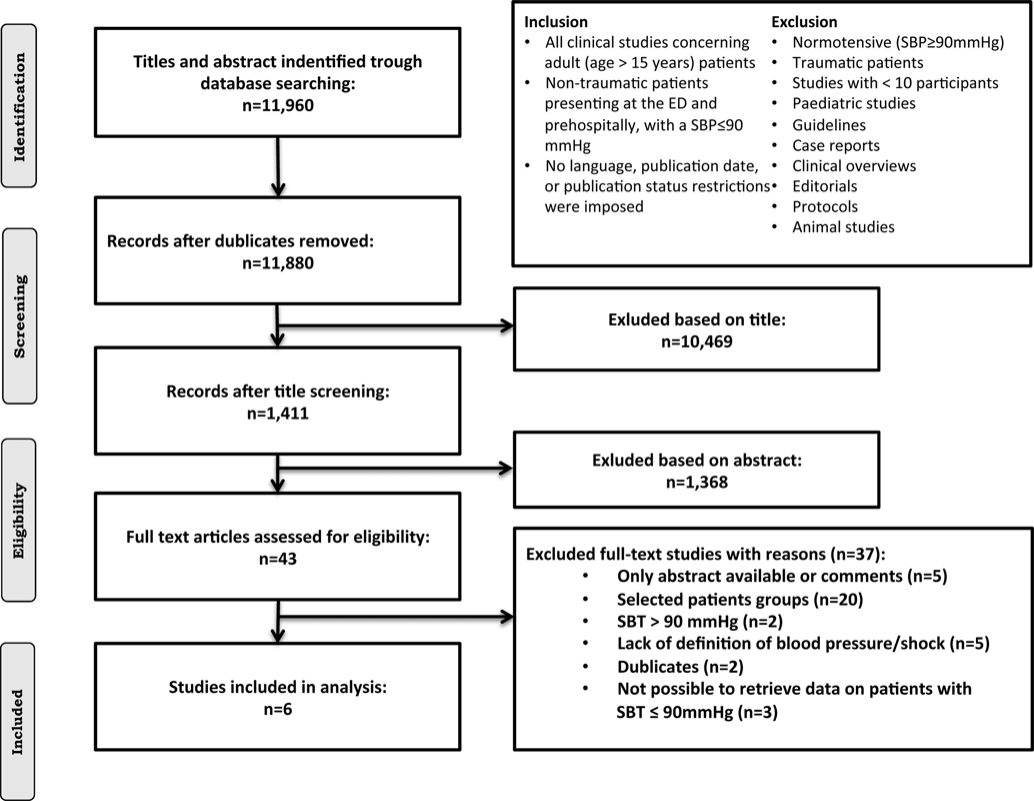
Inclusion and exclusion criteria and study flow for the systematic review.

We allowed inclusion of all study types that assessed prevalence, etiology or mortality as outcomes. Studies of higher evidence as RCTs were prioritized but also non-randomized trials and observational studies (cohort studies, case-control studies and cross-sectional studies) were considered. Studies with fewer than 10 patients, studies regarding children, animals or trauma were excluded as well as publications considered as editorials, clinical guidelines, comments or protocols. The review did not pose any restrictions on language, publication date or publication status. Inclusion and exclusion criteria are listed in Fig. 1.

### Information sources and search strategy

A search strategy was developed and tested by an experienced information scientist (*JW*, *see acknowledgments*). The literature search included Medical Subject Headings (MeSH), Emtree headings and related text and keyword searches in a manner that combined terms related to hypotension and shock in the ED and in the prehospital setting.

The following MESH-terms were used: (hypotension OR hypotensive OR shock OR low blood pressure) AND (prehospital OR emergency service hospital) AND (prognosis OR prognostic OR prevalence OR incidence OR mortality OR death rate OR etiology OR etiological OR epidemiology). Identification of studies were conducted by a computer-based systematic search on 8th August 2013 using MEDLINE (Ovid: 1966 to August, 2013), EMBASE (Ovid: 1974 to August, 2013), CINAHL (Ovid: 1981 to August 2013), DARE (Ovid:1990 to August, 2013) and the Cochrane Library (Ovid: August, 2013).

See S1 Search Strategy for details.

### Study selection

All studies were collated in an EndNote X5 bibliographic database (2011 Thomsen Reuters) by exporting citations from databases (PubMed, Embase etc.) directly into EndNote. After duplicates were removed, one researcher (JGH) reviewed studies on title level according to the eligibility criteria. Articles in any language that studied hypotensive or shocked patients in the ED or prehospital setting were included. Hereafter, eligibility assessment was performed independently and in duplicate in a blinded standardized manner by two reviewers (JGH and CNB) on the abstract level. After agreement, eligible abstracts were reviewed in full text. Disagreements between reviewers were resolved by consensus. If no agreement could be reached, a third author (ATL) would decide. Six studies fulfilled our eligibility criteria as they assessed adult patients with nontraumatic SBP ≤ 90 mmHg in the ED or prehospital setting. Finally, we conducted a hand-search of every eligible article in order to retrieve additional studies from the reference lists together with consulting experts within the field (CP and SM).

Reasons for excluding full-text articles were documented (see Fig. 1). In brief: studies with highly selected subgroups of patients who did not represent the broad group of nontraumatic hypotensive patients were not eligible for inclusion in our review [8–27]. Studies with a definition of hypotension of SBP ≤ 100 mmHg, were excluded from the review in accordance to our inclusion criteria [14, 28, 29]. However if a subgroup of hypotensive patients with SBP ≤ 90 mmHg were available we retrieved and included these data from the studies. Such data were available in three publications [30–32]. Moreover we excluded studies with lack of consistent definitions of SBP or shock [33–36].

### Data collection process and Data items

The following data were extracted independently and in duplicate in an unblinded standardized manner by two reviewers (JGH and CNB) using predefined data fields: Bibliographical data (author, year, country and name of indexed database); Population characteristics; Inclusion and exclusion criteria; Intervention; Outcome; Study design. Disagreements were resolved by consensus with a third author (ATL). See Table 1 for details.

**Table 1:** Characteristics of included studies.

Author, Year, (Location), Indexed source, *Study Design*, *[Reference Number]*	Study Setting and study period	Study population	Inclusion criteria	Exclusion criteria	Proportion of patients with SBT≤90 mmHg (N/1000)	Main findings	Symptoms and Etiology	NOS/STROBE Score
Jones et al. 2004 (USA) Embase, PubMed and Cinahl *Case-Control [32]*	Prehospital 4 months (1995–2000)	14,379 patients assessed by EMS, N = 273, SBT≤90 mmHg	Age > 17 years, SBT<100 mmHg during transport and 1 or more of 10 predefined symptoms of circulatory insufficiency	Trauma transports	19	Out of hospital hypotension showed higher inhospital mortality	*	7/19
Jones et al. 2006 (USA) Embase, PubMed and Cinahl *Cohort Study [30]*	Emergency Department 12 months (2004–2005)	113,000 patients assessed in the ED, N = 398, SBP≤90 mmHg	Age>17 years, SBP<100 mmHg and admission to the hospital from the ED	(1) Trauma in the past 24 hour, (2) Direct admission or transfer from another facility or no evaluation in the ED, (3) No vital signs measured	4	Hypotension showed increased risk of death during hospitalization	*	7/19
Merz et al. 2011 (Switzerland) Embase and Pubmed *Cohort Study [45]*	Emergency Department 7 months (2007–2008)	15,939 patients assessed in the ED, N = 202, SBP≤90 mmHg	Age>15 years, All ED patients	Patients treated on an outpatient basis	13	Vital signs abnormilities are independent predictors of hospital mortality	Etiology	5/19
Poloujadoff et al. 2006 (France) Embase and Pubmed Cohort Study *[46]*	Prehospital 12 months (2002–2003)	10,291 patients assessed by EMS, N = 131, SBP≤90 mmHg	Non-palpable radial pulse and unrecordable blood pressure at clinical presentation	Patients with cardiac arrest, arterial disease or acute limb ischaemia	9	Conditions associated with mortality; Cardiac arrest, Age, Glasgow Coma Scale	Etiology	6/12
Seymour et al. 2013 (USA) Embase, PubMed and Cinahl Cohort Study *[31]*	Prehospital 48 months (2002–2006)	154,644 patients assessed by EMS, N = 8,484, SBP≤90 mmHg	Nontraumatic, noncardiac arrest in whom a physical exam was performed by EMS personal	Age<18 years, Patients with missing SBP measurements, or SBP = 0 or SBP>300 mm Hg	19.5	SBP is a modest predictor of 30-day mortality	Etiology	8/20
Wang et al. 2011 (USA) Embase, PubMed and Cinahl Cross-sectional study *[44]*	Prehospital 36 months (2006–2008)	3,327,306 patients assessed by EMS, N = 39,424, SBP≤90 mmHg	SBP<80 mmHg, Special screening criteria designed for identification of shock	EMS without patient contact, Age<18 years, patients classified as dead on EMS arrival and cardiac arrest	9.5	39,424 (91.80%) presented with medical conditions and 3,517 (8.19%) with traumatic conditions	Symptoms	5/18

*Not assessed as objective in study.

SBP: Systolic Blood Pressure, EMS: Emergency Service Systems

### Risk of Bias in Individual Studies

In order to assess the quality of the observational studies we used the Newcastle-Ottawa quality assessment scale (NOS-scale) and the Strengthening the Reporting of Observational Studies in Epidemiology (STROBE) statement [37, 38]. The NOS scale assigns a maximum of 9 points for cohort studies and 8 points for case-control studies. Points are given for selection of participants and measurement of exposure as well as comparability of cohorts and assessment of outcomes and follow-up. Validity scores were evaluated as follows: ≤5, low quality; 6–7, medium quality; 8–9, high quality. The STROBE statement is a checklist of 22 items that provides general reporting recommendations for three main types of observational studies (cohort, case-control and cross-sectional studies). Descriptive results were presented as the crude number of patients included in the subgroups as well as proportions.

## Results

The literature search identified 11,960 articles. 1,711 articles were indexed in PubMed, 10,294 were indexed in Embase, 163 were indexed in Cinahl, 8 were indexed in DARE and 86 were indexed in the Cochrane Library. After duplicates were removed, 11,880 articles were evaluated for relevance. During the initial title screening process 10,469 articles were excluded. Further 1,368 articles were excluded after reading the abstracts, leaving 43 potentially relevant articles to be evaluated in full text together with a review of references in these articles. Ultimately, 6 articles were selected. Reasons for excluding full-text articles included three studies [14, 28, 29] with the definition of hypotension as 100 mmHg for inclusion without any analysis of a subgroup of 90 mmHg as well as five studies with no clear definition of hypotension or shock [33–36]. One publication was published as a comment [39] and four as conference abstracts with a selected population or it was not possible to retrieve data [40–43]. We excluded 20 studies as they assessed a selected group of patients in their analysis (e.g. trauma patients or patients with sepsis in the ED) [8–13, 15–27]. The flow diagram in Fig. 1 illustrates the study selection and explanation of the methods to obtain the final list of full-text articles.

### Study characteristics

Of the six studies included, four were indexed in PubMed, Embase and Cinahl [30, 31, 44]. The study by Merz et al. and Poloujadoff et al. were indexed in PubMed and Embase [45, 46]. None were indexed in the Cochrane Library or in DARE. All studies were published in English during the years 2004–2013.

All studies had an observational design with one cross-sectional, one case-control and four cohort studies conducted in Europe and USA. Four studies assessed prehospital patients [30, 31, 44, 46] and two studies assessed patients in the ED [30, 45]. The sample size of the selected studies ranged from 131 to 39,424 cases and enrolled a total of 48,912 cases with an age range of 18 to 88 years enrolled over a period of 4 months to 5 years. Women comprised 50–58% of the total participants in the studies. Table 1 shows all the studies with characteristics.

### Risk of bias within studies

By means of the STROBE checklist, the general reporting recommendations were in part followed among the studies with values from 12 to 20. The studies showed in general problems with follow-up and missing data for each variable of interest. The quality of the studies were in general considered low [44, 45] to moderate [32, 46] and ranged from 5–7 points according to the NOS-scale (see Table 1). One study was judged as a high quality study [31].

### Results of Individual Studies

#### Prevalence

**Fig 2:**
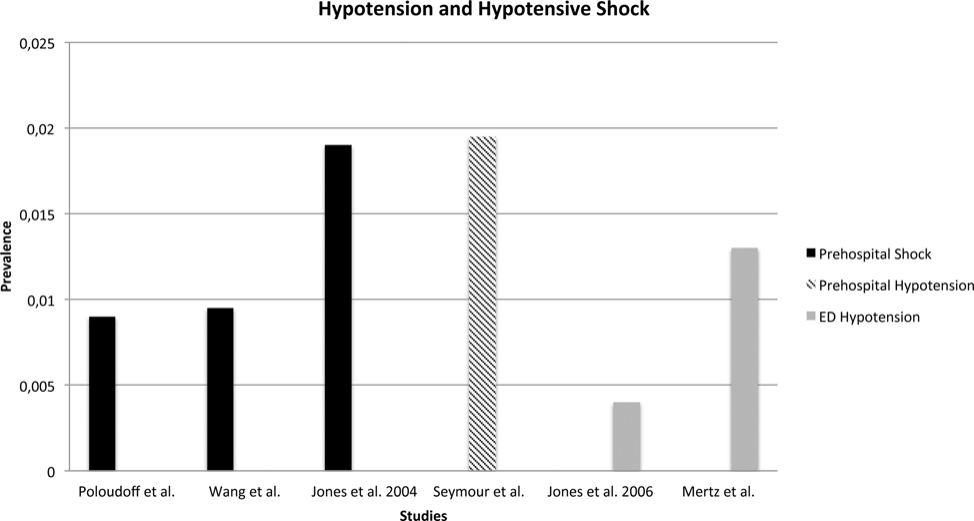
Prevalence of hypotension and hypotensive shock based on setting.

**Fig 3:**
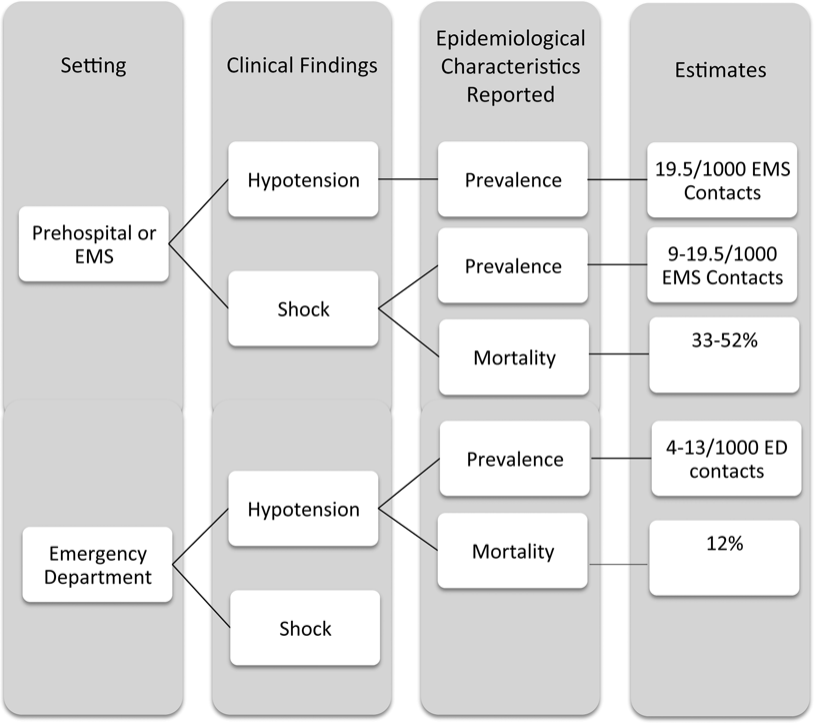
Visual overview of the clinical epidemiological characteristics reported based on setting and clinical findings.

It was possible to retrieve data on prevalence of all six studies (see Fig. 2 and Fig. 3). From data in the study by Seymour et al. we estimated a prevalence of prehospital hypotension of 19.5/1000 EMS contacts [31]. Three of the six included studies assessed the prevalence of hypotensive shock in the prehospital setting and reported a proportion of 9/1000 EMS contacts (Poloudoff et al.), 9.5/1000 EMS contacts (Wang et al.) and 19/1000 EMS contacts (Jones et al. 2004) [32, 44, 46]. Data among patients with SBP ≤ 90 mmHg in the ED gave an estimated prevalence of hypotension of 4–13/1000 ED contacts in the studies by Jones et al. (2006) and Merz et al. [30, 45]. Prevalence of shock in the ED was not available.

#### Etiology

It was not possible to compare etiological characteristic among any of the six eligible studies. Data on etiological characteristics were presented in four studies. In the prehospital setting, data were presented as types of shock (e.g. cardiogenic and septic) [46] or as suspected illnesses (e.g. dizziness, pain, dehydration) [44]. A third study used clinical specialties to present possible etiological characteristics (e.g. cardiology, neurology) [31]. In the ED setting, one study presented data as reasons for primary admission to the ED [45].

#### Mortality

**Fig 4:**
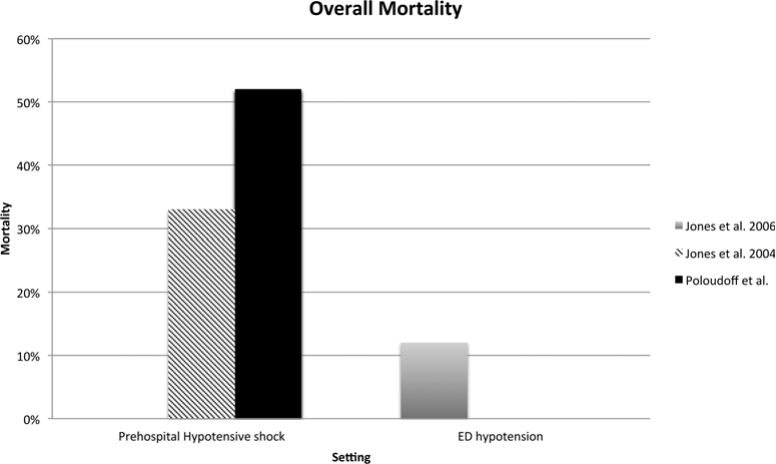
In-hospital mortality based on setting and presence of hypotension (Jones et al. 2006) and hypotensive shock (Poloudoff et al. and Jones et al. 2004).

It was possible to retrieve data on mortality from three studies (see Fig. 3 and Fig. 4) [30, 32, 46]. The inhospital mortality of patients already recognized with shock prehospitally was 33% to 52% [32, 46]. No data were available regarding patients suffering hypotension in the prehospital setting. Among hypotensive patients in the ED the inhospital mortality was 12% [30]. It was not possible to retrieve information on mortality regarding shock in the ED.

### Synthesis of Results

The retrieved data did not provide any results that were feasible for the conduction of a meta-analysis due to substantial heterogeneity between the studies.

## Discussion

This systematic review of epidemiological characteristics of unselected adults with hypotension of nontraumatic origin with or without shock in the ED and the prehospital setting identified six studies eligible for analysis. The studies were all observational with four studies assessing patients in the prehospital setting and two studies assessing patients in the ED setting. The data showed a rather consistent pattern of the prevalence of prehospital hypotension and hypotensive shock, with high inhospital mortality rates among patients suffering both hypotension and shock detected in the prehospital setting compared to those reported to have hypotension after their arrival in the ED. Data on prevalence and mortality among patients suffering shock in the ED was not available. Furthermore the studies showed a diversified picture of the causal mechanism underlining lack of evidence to arrive a definite conclusion on the etiological characteristics.

We were only able to include six observational studies. It might be argued that the data at hand is a result of a rigorous set of eligibility criteria, however more than 11,000 individual research papers met our literature search which suggest a broad search strategy. Based on these findings, the overall conclusion is that epidemiological characteristics among undifferentiated hypotensive patients have not been widely addressed in the general ED and prehospital populations despite the common presentation of patients.

Jones et al. have conducted several studies on nontraumatic hypotensive patients, defined as a SBP of ≤ 100 mmHg, challenging the traditional definition of hypotension [28, 30, 32, 47]. These studies conclude a “dose-response” relationship between the duration of hypotension and the adverse outcome beginning at SBP ≤ 100 mmHg and enlightens the fact that unselected hypotension (defined as SBP ≤ 100 mmHg) is a common condition in the ED and the prehospital setting and a strong predictor of inhospital mortality. The study by Seymour et al. also questions the traditional definition of hypotension as (SBP ≤ 90 mmHg) as a large proportion of patients are misclassified in relation to outcome [31]. Observational studies in traumatic hypotensive populations in the ED and prehospital setting also advocate for a higher threshold of SBP as a similar pattern has been detected regarding changes in mortality related to the level of SBP [10, 25, 48].

In our inclusion criteria for hypotension we chose to use a SBP ≤ 90 mmHg as this threshold has been a widely accepted definition of hypotension as well as a traditional alert parameter in many clinical guidelines and triage systems involving critically ill patients [3, 4]. Perhaps this threshold will change in the future.

Although the majority of critically ill patients are identified and initially treated in the prehospital and ED setting the superiority of the research conducted have been limited to selected patient populations in the ICU´s and specialized units [12, 49–51]. Since undifferentiated hypotensive patients in the emergency department setting frequently presents with heterogeneous symptoms and pathophysiology, studying a larger group of these patients is often challenging. As a result, the recruitment of patients suffering circulatory failure or hypotension are often identified in the emergency setting if certain highly selected eligibility criteria are fulfilled before entering the study for further analysis. These studies give valid information on the outcome of certain subgroups of patients with clarified etiologies (e.g. sepsis, acute myocardial infarction) but also reflect the specialized focus on these patient groups. Furthermore, it highlights the need of further knowledge of the undifferentiated hypotensive and critically ill patient populations in the ED and prehospital setting in order help the acute clinical personnal improve the ability to identify, prioritize and allocate resources as well as improve patient outcome in these settings.

### Strengths and Limitations

This systematic review did not pose any restrictions on language, publication date or type of study in our search terms and therefore should not suffer any language or publication bias. We consulted an experienced information scientist who peer-reviewed our MeSH terms and helped construct our literature search prior to the conduction of the search. Also our eligibility assessment was performed independently, in duplicate and in a blinded standardized manner by two reviewers in order to minimize bias.

In the identified studies the accuracy of systolic blood pressure measurements by auscultation and automatic oscillometric devices might be low, especially when assessed in the prehospital setting due to noise, moving vehicles etc. Although we were unable to validate whether blood pressure measurements by auscultation or automatic oscillometric devices are correctly used or calibrated in the included studies these are the conditions that apply for any assessment being performed in the prehospital environment.

As evident by the results, the eligible studies did not add sufficient data for the conduction of a statistical analysis (including meta-analysis). We interpret these findings as a reflection of the substantial heterogeneity and varied quality across the studies, as well as the lack of research on the topic.

## Conclusion

There is inadequate knowledge about the patient presenting with nontraumatic hypotension or shock in the ED or prehospital setting. The available studies suggest that 2% of EMS contacts present with nontraumatic hypotension and 1–2% with shock. The inhospital mortality of shock is 33–52%. ED prevalence of hypotension is 4–13/1000 contacts with an inhospital mortality of 12%.

The prevalence, etiological characteristics and mortality of shock in the ED are not well described.

## Supporting Information

S1 Protocol (DOC)

S1 Checklist (DOC)

S1 Search Strategy (DOCX)
