# Supplementary material for: Nontraumatic Hypotension and Shock in the Emergency Department and the Prehospital setting, Prevalence, Etiology, and Mortality: A Systematic Review
Source: PLoS One. 2015 Mar 19;10(3):e0119331. doi: 10.1371/journal.pone.0119331 (PMC4366173; doi:10.1371/journal.pone.0119331)
Supplement: S1 Protocol — (DOC) [file pone.0119331.s001.doc]

**S1 Protocol**

**Research protocol for systematic review**

Title: Non-traumatic hypotension and Shock in the Emergency Department and the prehospital setting; Prevalens, Etiology and Mortality; a systematic review.

Journal: Open-access journal

Authors:

1. Jon Holler MD, Emergency Department, Odense University Hospital and University of Southern Denmark
2. Camilla Bech MD, Emergency Department, Odense University Hospital and University of Southern Denmark
3. Søren Mikkelsen MD, Department of Anaesthesiology and Intensive Care Medicine,, Odense University Hospital and University of Southern Denmark.
4. Daniel Pilsgaard Henriksen MD, Emergency Department, Odense University Hospital and University of Southern Denmark
5. Court Pedersen MD, DMSci professor, Department of Infectious Disease, Odense University Hospital and University of Southern Denmark.
6. Annmarie Lassen MD, phd, DMSci professor, Emergency Medicine Odense University Hospital and University of Southern Denmark

Rationale:

To our knowledge, no systematic review has ever been conducted on non-traumatic hypotension and shock prehospitally and in the emergency department, despite the mortality and common presentation.

In order to clarify the level of evidence regarding prevalence, etiology and mortality of non-traumatic hypotensive shock we will conduct a systematic review on the topic.

Objectives:

Purpose and intended goals of the literatur review:

- To evaluate the level of evidence regarding the prevalens, etiology and mortality of non-traumatic hypotension in the ED and in the prehospital setting.

We will use PICOS criteria to improve the explicitness of the review questions:

Participants: Adult hypotensive (SBP ≤90 mmHg) patients with- and without shock in the acute setting, i.e. patients assessed pre-hospitally or in the ED.

Intervention/exposure: All interventions regarding patients with hypotension or shock.

Comparisons: Hypotensive patients in any control group with - and without shock receiving interventional treatment or standard of care treatment.

Outcome: Prevalence, Aetiology and Mortality.

Study design: Randomized controlled trials (RCTs) and observational studies (cohort studies, case-control studies and cross-sectional studies).

**Methods**

Protocol and registration:

Our protocol and systematic review is conducted according to the PRISMA statement.

Eligibility criteria:

Inclusion

- All clinical studies concerning adult (age > 15 years) patients.
- Non-traumatic patients presenting at the ED and prehospitally, with a SBP≤90 mmHg
- No language, publication date, or publication status restrictions are imposed.

Exclusion

- Normotensive (SBP≥90mmHg)
- Traumatic patients
- Studies with < 10 participants
- Paediatric studies
- Guidelines
- Case reports
- Clinical overviews
- Editorials
- Protocols
- Animal studies

Information sources:

Pubmed, EMBASE, Cochrane Library and CINAHL Additional studies are identified by consulting clinical experts (SM/CP).

Criteria for the practical electronic search strategy:

Literature search will be conducted together with a experienced information scientist.

Study selection:

Eligibility assessment is performed independently in an unblinded standardized manner by 2 reviewers (JGH and CB). Disagreements between reviewers are resolved by consensus. If no agreement can be reached a third author (AL/SM) will decide.

Data collection process:

Independent extraction of articles by 2 authors (JGH and CB) using predefined data fields, including study quality indicators.

**Results:**

Depending on results, consider:

- Meta-analysis.
- Descriptive analysis

**Discussion:**

Summary of evidence

Limitations

Conclusions including implications for future research

**References**

1. Liberati, A., et al.*, The PRISMA statement for reporting systematic reviews and meta-analyses of studies that evaluate health care interventions: explanation and elaboratio*n. PLoS Med, 2009**.** 6(7): p. e1000100.
