# Supplementary material for: Nontraumatic Hypotension and Shock in the Emergency Department and the Prehospital setting, Prevalence, Etiology, and Mortality: A Systematic Review
Source: PLoS One. 2015 Mar 19;10(3):e0119331. doi: 10.1371/journal.pone.0119331 (PMC4366173; doi:10.1371/journal.pone.0119331)
Supplement: S1 Search Strategy — (DOCX) [file pone.0119331.s003.docx]

**S1 Search Strategy**

**Medline:** *Date of search; 8th August 2013, number of hits; 1,711*

Pubmed was systematically search using the the following Medical Subject Headings (MeSH),

(("hypotension"[MeSH Terms] OR "hypotension"[All Fields]) OR hypotensive[All Fields] OR ("shock"[MeSH Terms] OR "shock"[All Fields]) OR ("hypotension"[MeSH Terms] OR "hypotension"[All Fields] OR ("low"[All Fields] AND "blood"[All Fields] AND "pressure"[All Fields]) OR "low blood pressure"[All Fields])) AND (prehospital[All Fields] OR ("emergency service, hospital"[MeSH Terms] OR ("emergency"[All Fields] AND "service"[All Fields] AND "hospital"[All Fields]) OR "hospital emergency service"[All Fields] OR ("emergency"[All Fields] AND "service"[All Fields] AND "hospital"[All Fields]) OR "emergency service hospital"[All Fields])) AND (("prognosis"[MeSH Terms] OR "prognosis"[All Fields]) OR prognostic[All Fields] OR ("epidemiology"[Subheading] OR "epidemiology"[All Fields] OR "prevalence"[All Fields] OR "prevalence"[MeSH Terms]) OR ("epidemiology"[Subheading] OR "epidemiology"[All Fields] OR "incidence"[All Fields] OR "incidence"[MeSH Terms]) OR ("mortality"[Subheading] OR "mortality"[All Fields] OR "mortality"[MeSH Terms]) OR ("mortality"[Subheading] OR "mortality"[All Fields] OR ("death"[All Fields] AND "rate"[All Fields]) OR "death rate"[All Fields] OR "mortality"[MeSH Terms] OR ("death"[All Fields] AND "rate"[All Fields]) OR "death rate"[All Fields]) OR ("etiology"[Subheading] OR "etiology"[All Fields] OR "causality"[MeSH Terms] OR "causality"[All Fields]) OR etiological[All Fields] OR ("epidemiology"[Subheading] OR "epidemiology"[All Fields] OR "epidemiology"[MeSH Terms]))

**EMBASE (1950 - 2013):** *Date of search; 8th August 2013, number of hits; 10,294*
***-*** *Search terms used:*

- anaphylactic shock
- blood
- burn shock
- cardiogenic shock
- death
- death rate
- electric shock
- emergency
- emergency care
- emergency health service
- emergency service hospital
- epidemiology
- etiological
- etiology
- follow up
- heart infarction
- hemorrhagic shock
- hospital
- human
- hypotension
- hypotensive
- hypovolemic shock
- incidence
- low
- low blood pressure
- mortality
- physical disease by etiology and pathogenesis
- prehospital
- pressure
- prevalence
- prognosis
- prognostic
- rate
- resuscitation
- septic shock
- service
- shock

standardized incidence

ratio

**Search Returned:**

  10,294 results

Search key

1. exp hypotension/

2. hypotensive.mp.

3. shock.mp. or exp electric shock/ or exp cardiogenic shock/ or exp hypovolemic shock/ or exp heat shock response/ or exp burn shock/ or exp septic shock/ or exp anaphylactic shock/ or exp toxic shock syndrome/ or shock/ or exp hemorrhagic shock/ or exp toxic shock syndrome toxin 1/

4. low blood pressure.mp. or exp hypotension/

5. exp resuscitation/ or exp emergency health service/ or exp heart infarction/ or prehospital.mp. or exp emergency care/

6. emergency service hospital.mp. or exp emergency health service/

7. exp prognosis/ or prognosis.mp.

8. prognostic.mp. or exp prognosis/ or exp follow up/

9. prevalence.mp. or prevalence/

10. exp incidence/ or exp standardized incidence ratio/ or incidence.mp.

11. death rate.mp. or exp mortality/

12. etiology.mp. or exp etiology/

13. etiology.mp. or exp etiology/ or "physical disease by etiology and pathogenesis"/

14. etiological.mp.

15. exp epidemiology/ or epidemiology.mp.

16. 1 or 2 or 3 or 4

17. 5 or 6

18. 7 or 8 or 9 or 10 or 11 or 12 or 13 or 14 or 15

19. 16 and 17 and 18

20. exp mortality/ or exp epidemiology/ or exp cohort analysis/ or cohort.mp. or exp follow up/

21. 19 and 20

22. human/

23. human/

24. exp adolescence/ or exp adolescent disease/ or adolscent.mp.

25. adult/

26. 19 and 22 and 24

27. 19 and 22

28. nontraumatic.mp.

29. 19 and 28

30. shock.mp. or exp electric shock/ or exp cardiogenic shock/ or exp hypovolemic shock/ or exp burn shock/ or exp septic shock/ or exp anaphylactic shock/ or exp shock/ or exp hemorrhagic shock/

31. 1 or 2 or 4 or 30

32. 17 and 18 and 31

33. 23 and 32

**COCHRANE:** *Date of search; 8th August 2013, number of hits; 86*

(hypotension OR hypotensive OR shock OR low blood pressure) AND (prehospital OR emergency service hospital) AND (prognosis OR prognostic OR prevalence OR incidence OR mortality OR death rate OR etiology OR etiological OR epidemiology).

**Other sources:**

**CINAHL (Ovidsp)**

*Date of search; 8th August 2013, number of hits; 163*

(hypotension OR hypotensive OR shock OR low blood pressure) AND (prehospital OR emergency service hospital) AND (prognosis OR prognostic OR prevalence OR incidence OR mortality OR death rate OR etiology OR etiological OR epidemiology).
